# Genome-Wide Identification of the WD40 Gene Family and Functional Analysis of a Candidate Gene Regulating Seed Quality in Soybean

**DOI:** 10.3390/genes17080904

**Published:** 2026-07-30

**Authors:** Hui Chen, Sunlei Ding, Haiyan Bi, Qimike Shan, Xiaolei Shi, Bingbing Lei, Zhigang Liu, Yangyang Yang, Rui Tian, Yongliang Yan

**Affiliations:** 1 Xinjiang Key Laboratory of Crop Biotechnology, Biological Breeding Laboratory, Academy of Agricultural Sciences of Xinjiang Uyghur Autonomous Region, Urumqi 830091, China; chenhui001103@163.com (H.C.); liuzg1850@163.com (Z.L.); 2 Institute of Crop Research/National Central Asian Characteristic Crop Germplasm Resources Medium-Term Gene Bank (Urumqi), Academy of Agricultural Sciences of Xinjiang Uyghur Autonomous Region, Nanchang Road 403, Urumqi 830091, China; dsl_1421013285@163.com (S.D.); shanqimike08@126.com (Q.S.); xl19961125@163.com (X.S.); bingbingl7828@163.com (B.L.); xiaoyangxiaoenyang@163.com (Y.Y.); 3 Xinjiang Uyghur Autonomous Region Agricultural Technology Extension General Station, Shengli Road 157, Urumqi 835099, China; bihaiyan1026@163.com

**Keywords:** GmWD40 family, *GmWD40-257*, EMS, quality traits, AlphaFold 3

## Abstract

**Background**: Soybean is an important crop with multiple uses for oil, food, and feed, providing 50% of the vegetable protein and 20% of the edible oil in the world. The WD40 family genes play crucial regulatory roles in growth, development, secondary metabolism, and stress responses. However, the definition of WD40 family genes in soybean remained unclear, which limited their application potential in genetic improvement. **Methods**: To identify soybean WD40 family members and screen candidate genes for breeding improvement, this study performed genome-wide identification of the soybean WD40 gene family via bioinformatic approaches based on the latest Williams 82 reference genome (Wm82.a6.v1). Meanwhile, the function of the family gene *GmWD40-257* regulating seed quality was analyzed. **Results**: The results showed that a total of 458 *GmWD40* genes were identified, which were distributed on the 20 chromosomes. Subcellular localization showed that most members were mainly concentrated in the nucleus, chloroplast, and cytoplasm. Phylogenetic tree analysis divided the 458 *GmWD40* genes into eight groups. Synteny analysis identified 160 syntenic genes between soybean and *Arabidopsis thaliana*. Conserved motif analysis identified ten core motifs. The promoter regions of *GmWD40* contained 19 types of cis-acting elements. Functional analysis revealed that the nonsense mutation of *GmWD40-257* significantly reduced the content of oil, palmitic acid, oleic acid, linoleic acid, α-linolenic acid and soluble sugar, while significantly increasing the contents of protein, γ-tocopherol and δ-tocopherol. **Conclusions**: A total of 458 members of the WD40 gene family were identified in soybean. Among these, *GmWD40-257* was found to positively regulate the contents of soybean oil, palmitic acid, oleic acid, linoleic acid, α-linolenic acid and soluble sugar, while negatively regulating the contents of soybean protein, γ-tocopherol and δ-tocopherol.

## 1. Introduction

Soybean (*Glycine max* (L.) Merr.), the fourth most important crop in the world, was domesticated from its wild relative *Glycine soja* [Sieb. and Zucc.] in China during the Zhou dynasty around 5000~6000 years ago [1,2]. As a nutrient-dense crop, soybean provided protein, oil, carbohydrates, minerals, vitamins, folic acid, dietary fiber, isoflavones and other essential nutrients for both human food and animal feed. Benefiting from its irreplaceable nutritional and economic value, global soybean production has expanded dramatically over the past decades [3,4].

WD40 proteins, also known as WD40 repeat proteins, are a superfamily in eukaryotes, playing a central role in orchestrating protein–protein interactions [5]. WD40 proteins carry a highly conserved WD40 motif, which contains 40~60 amino acid residues, starting with glycine (G)-histidine (H) and ending with tryptophan (W)-aspartate (D) dipeptide [6,7,8,9,10]. In plants, WD40 proteins are widely involved in various processes such as growth, development and secondary metabolite accumulation [11,12,13,14].

WD40 proteins act as key regulators of gibberellic acid (GA) and abscisic acid (ABA) signaling pathways during seed germination. In *A. thaliana*, WD40 protein RIE1 (RGL2-interacting E3 ligase 1) interacted with RGL2 (RGA-like 2) and affected the stability of the RGL2 protein, thereby participating in GA-mediated regulation of seed dormancy and germination [15]. Furthermore, overexpression of *ABT* (ABA Signaling Terminator, encoding a WD40 protein) promoted seed germination under ABA treatment, whereas knockout of *ABT* produced the opposite effect. Mechanistic analysis revealed that ABT interacted with PYR1/PYL4 (Pyrabactin Resistance 1/PYR1-Like 4) and ABI1/ABI2 (Abscisic Acid Insensitive 1/2) proteins. This binding disrupted the interaction between PYR1/PYL4 and ABI1/ABI2, and further reduced the inhibitory effect of PYR1 on the phosphatase activity of ABI1/ABI2. As a result, ABI1/ABI2 suppressed SnRK2 (SNF1-related protein kinase 2) autophosphorylation and terminated ABA signaling. These changes attenuated ABA responses and ultimately promoted seed germination and seedling establishment [16].

Similarly, WD40 protein RUP1 and RUP2 (REPRESSOR OF UV-B PHOTOMORPHOGENESIS 1 and 2) also played important roles in ABA signaling to regulate seed germination and early seedling development in *A. thaliana*. Loss of *RUP1* and *RUP2* function impaired germination and establishment under ABA stress. RUP1 and RUP2 interacted with ABI5 (Abscisic Acid Insensitive 5) to promote ABI5 ubiquitination and degradation, whereas ABI5 negatively regulated *RUP1* and *RUP2* expression [17]. Moreover, mutation of the WD40 gene *XIW1* (encoding XPO1-Interacting WD40 protein 1) reduced the expression of ABI5 and ABA-responsive genes under salt treatment in *A. thaliana*, thereby increasing seed germination in the mutant [18]. In addition, WD40 repeat protein GTS1 (GIGANTUS1) also negatively regulated seed germination in *A. thaliana*, which is highly expressed during the embryo development stage [19].

Furthermore, WD40 proteins have been shown to modulate seed development through diverse mechanisms in plants. The overexpression of the *Spartina alterniflora* WD40 gene *SaTTG1* in *A. thaliana* resulted in early flowering and increased seed size [20]. In maize, WD40 protein SHREK1 (Shrunken and Embryo Defective Kernel 1) was highly accumulated in developing seed, and loss of *SHREK1* function resulted in embryogenesis abortion, storage reserves diminution, and downregulation of the expression of genes associated with developmental processes, carbohydrate catabolic processes, and nutrient reservoir activity [21]. In contrast, several WD40 proteins acted as negative regulators in seed development. In maize, selection in the noncoding upstream regions reduced *KRN2* (Kernel Row Number 2, encoding WD40 protein) expression, leading to an increase in grain number through an increase in kernel rows, whereas in rice, *OsKRN2* (encoding WD40 protein) negatively regulated grain number by controlling secondary panicle branches [22]. In *A. thaliana*, the WD40 protein AtTTG1 (TRANSPARENT TESTA GLABRA 1) was involved in the accumulation of seed storage reserves. The mutation of *AtTTG1* exhibited increased embryo dry weight, as well as higher levels of starch, total protein, and fatty acids, and mechanistic analysis suggested that *AtTTG1* negatively regulated the accumulation of seed storage reserves by repressing the expression level of 2S3 encoding a 2S albumin precursor [23].

Moreover, WD40 proteins also regulated the accumulation of plant secondary metabolites by forming the MYB-bHLH-WD40 (MBW) complex with MYB (Myeloblastosis) and bHLH (Basic Helix-Loop-Helix). This regulatory module was exemplified across various species. In rice, the WD40 gene *OsTTG1* was responsive to light and temperature, and the OsTTG1 mutant showed significantly decreased anthocyanin accumulation in various rice organs [24]. OsTTG1 interacted with OsbHLH148 and OsMYBS3 to form an MBW complex that directly promoted the expression of both *OsANS* (involved in anthocyanidin biosynthesis) and the cold-stress-responsive transcription factor *OsDREB1*, and simultaneously coordinated anthocyanin synthesis and cold adaptation [25]. In *Camellia sinensis*, CsWD40 interacted with two bHLH transcription factors and two MYB transcription factors. Ectopic expression of *CsWD40* alone in tobacco led to a significant increase in anthocyanin levels, whereas coexpression of *CsWD40* and *CsMYB5e* elevated both anthocyanin and proanthocyanidin (PA) levels [26]. This synergistic mechanism was further validated in *Raphanus sativus*, where co-expression of *RsTTG1* with *RsMYB1* and *RsTT8* in tobacco activated the transcription of anthocyanin biosynthetic genes, resulting in enhanced anthocyanin accumulation [27]. Besides anthocyanins, WD40 proteins also regulated phenolic metabolites. In *Medicago truncatula*, deficient expression of *MtWD40-1* impaired the accumulation of mucilage and a range of phenolic compounds (PAs, epicatechin, other flavonoids, and benzoic acids) in the seed, while reducing epicatechin levels in flowers and decreasing isoflavone levels in roots [28].

Current knowledge regarding the biological roles of WD40 proteins in soybean remains limited, with most existing studies centering on the regulation of secondary metabolism. Glyma.06G136900 (GmWD40) formed a ternary regulatory complex together with the bHLH transcription factor GmTT8 (TRANSPARENT TESTA 8) and the MYB regulators GmTT2A/GmTT2B, and this assembled complex strongly activated the promoters of two key genes, *GmANR1* and *GmLAR2*, which encoded anthocyanidin reductase and leucoanthocyanidin reductase, respectively [29]. In addition, Glyma.06G136900 served as a molecular scaffold stabilizing the GmTT8-GmMYB115 heterodimer, which promoted the transcription of anthocyanin biosynthesis-related gene *GmANS* yet repressed transcription of the isoflavone biosynthesis gene *GmIFS*, thereby balancing metabolic flux from shared flavonoid precursors [30]. Another soybean WD40 protein, GmWD40-7 (Glyma.19G170400), interacted with GmMYB12B2 and GmbHLH13 to assemble another MBW complex, increasing the expression of *GmCHS7*, which encoded chalcone synthase [31]. Beyond secondary metabolism, WD40 proteins participate in soybean growth, stress tolerance and disease resistance. The WD40 protein PH13 (Plant Height 13, Glyma.13G276700) positively modulated soybean plant height. It interacted with GmCOP1 (Constitutive Photomorphogenic 1) to drive the degradation of STF1/2 (TGACG-Motif Binding Factor 1/2) transcription factors, which repressed stem elongation, and consequently accelerated exaggerated stem elongation induced by long-day photoperiods and low blue light. Conversely, loss of *PH13* function significantly improved soybean shade tolerance and lodging resistance [32]. The WD40-repeat protein GmPRL1b (PLEIOTROPIC REGULATORY LOCUS 1b) interacted with the NAC (NAM-ATAF-CUC) transcription factor GmST2 (SALT TOLERANT 2), and elevated the accumulation of GmST2 protein via post-translational regulation. As an upstream regulator, GmPRL1b activated the jasmonic acid biosynthesis pathway, thereby enhancing soybean tolerance to salt stress and resistance to *Botrytis cinerea* [33].

WD40 gene families have been identified in other plants, while the characterization of soybean WD40 family members remained unclear, which limited their potential for genetic improvement. In view of this, the soybean WD40 gene family was identified at the whole-genome level based on the latest reference genome of Williams 82 (Wm82.a6.v1), and its physicochemical properties, chromosomal location, gene structure, motif composition, and phylogenetic relationships were systematically analyzed. In addition, the function of *GmWD40-257* was analyzed using a nonsense mutation induced by EMS (Ethyl methanesulfonate). This study provided a theoretical foundation for further investigating the functions of the WD40 gene family in soybean and offered novel insight into the mechanisms underlying soybean quality-related traits.

## 2. Materials and Methods

### 2.1. Materials

The EMS-induced nonsense mutation *Gmwd40-257* of Williams82 and wild type (WT) were provided by Professor Qingxin Song of Nanjing Agricultural University [34].

### 2.2. Identification of the GmWD40 in Soybean

GmWD40 family members were systematically identified based on *A. thaliana* WD40 proteins. The sequences of WD40 proteins in *A. thaliana* were downloaded from the Phytozome database (https://phytozome-next.jgi.doe.gov/, accessed on 12 January 2026) [9,35]. The latest version of the soybean Williams 82 genome (Wm82.a6.v1) was obtained from the SoyBase database (https://www.soybase.org/, accessed on 12 January 2026) [36]. Subsequently, the WD40 proteins of *A. thaliana* were used as query sequences to identify homologous WD40 proteins in soybean via BLAST search in TBtools software v2.487, with an E-value cutoff of <1 × 10^−5^ [37]. To confirm the presence of WD40 domains in the identified proteins and the absence of duplicate proteins, additional domain analysis was conducted using multiple databases, including Pfam (PF00400, http://pfam.xfam.org/, accessed on 12 January 2026) and SMART (https://smart.embl.de/, accessed on 12 January 2026) [38,39]. The physicochemical properties of GmWD40, including amino acid number, molecular weight and isoelectric point (pI), were calculated using TBtools v2.487 [37]. Subcellular localization predictions for GmWD40 were performed using the WoLF PSORT tool (https://wolfpsort.hgc.jp/, accessed on 12 January 2026) [40].

### 2.3. Promoter Analysis of GmWD40 in Soybean

The promoter sequences (2000 bp upstream) of GmWD40 were extracted from Phytozome (https://phytozome-next.jgi.doe.gov/, accessed on 15 January 2026), which were analyzed for cis-regulatory elements using the PlantCARE database (http://bioinformatics.psb.ugent.be/webtools/plantcare/html/, accessed on 15 January 2026) [41]. TBtools software v2.487 was employed to visualize cis-regulatory elements [37].

### 2.4. Phylogenetic and Synteny Analysis of GmWD40

The Phylogenetic tree of GmWD40 proteins, tomato WD40 proteins and *A. thaliana* WD40 proteins was constructed using MEGA 11.0 software with the Maximum Likelihood (ML) method and a bootstrap value of 1000 to assess the reliability of the branches and the resulting tree was visualized using iTOL (https://itol.embl.de/itol.cgi, accessed on 30 January 2026) [42,43]. To assess the evolutionary relationships, GmWD40 proteins were aligned with the WD40 proteins from *A. thaliana* using TBtools software v2.487 [37].

### 2.5. Analysis of Gene Structure, Conserved Motifs, and Chromosome Location of GmWD40

Gene structure analysis of GmWD40 was performed using TBtools software v2.487 [37]. Conserved motifs of the GmWD40 proteins were analyzed on the MEME 5.5.9 (https://meme-suite.org/meme/tools/meme, accessed on 22 January 2026), with the maximum number of motifs set to 10 [44]. The chromosome location map, conserved motifs and gene structures of GmWD40 were visualized in TBtools software v2.487 [37].

### 2.6. Analysis of Protein Interaction Network for GmWD40

Protein interaction network analysis of the GmWD40 genes was conducted using STRING 12.0 (https://cn.string-db.org/cgi/input?sessionId=bWyfZNxORAJb&input_page_active_form=multiple_sequences, accessed on 26 January 2026), with the minimum required interaction score set to 0.9 [45].

### 2.7. Expression Analysis of GmWD40

To characterize the expression levels of GmWD40 family members throughout soybean seed development, we utilized published transcriptome data of soybean seed development (accession GSE99571), which was downloaded from the NCBI GEO database (https://www.ncbi.nlm.nih.gov/geo/query/acc.cgi?acc=GSE99571, accessed on 24 July 2026).

### 2.8. Functional Analysis of GmWD40-257 in Soybean Seed Quality Traits

**Figure 1:**
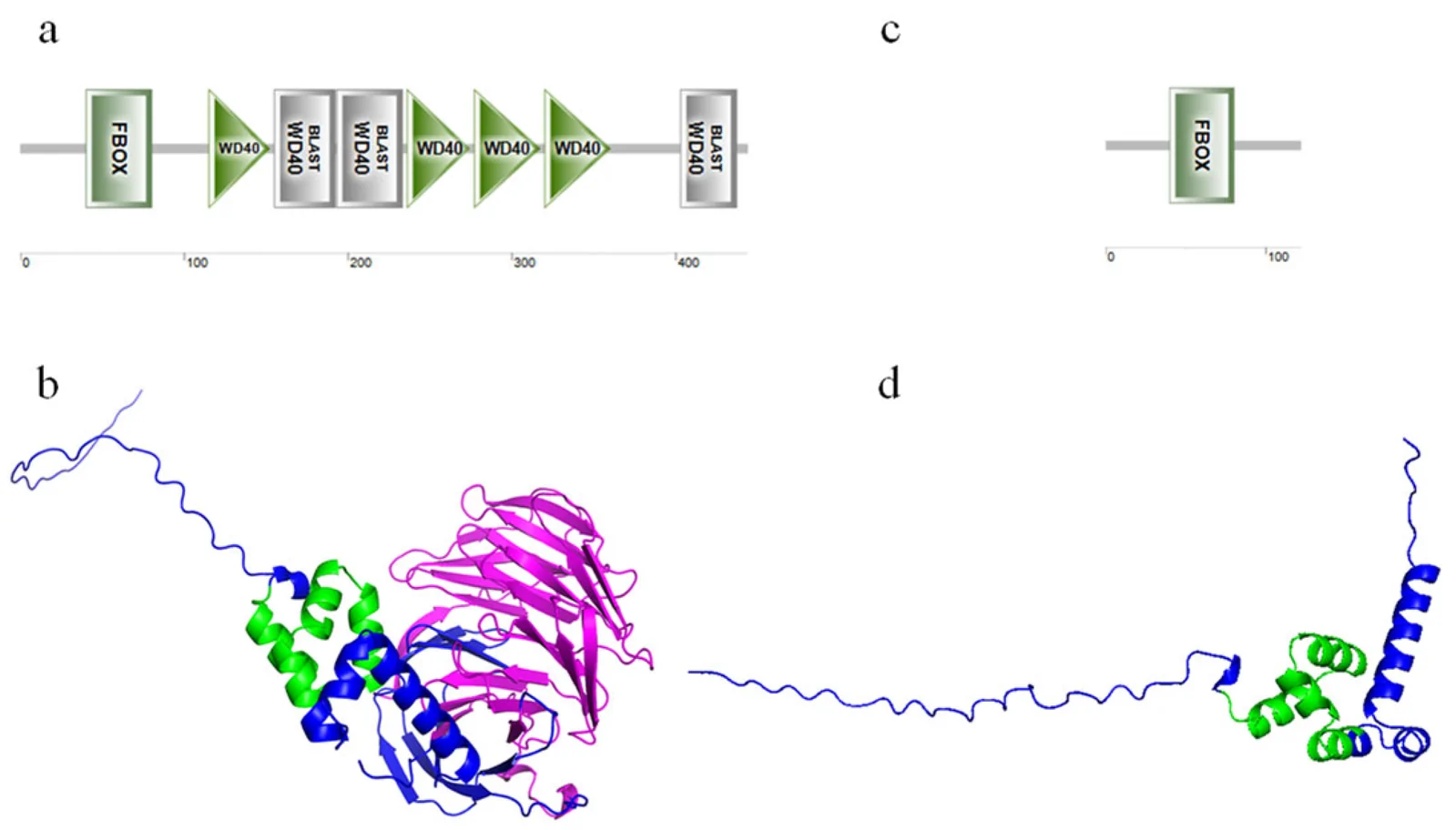
The protein structure of wild-type and nonsense mutation of *GmWD40-257* used in the present study. (**a**) The schematic diagram of the conserved domain for *GmWD40-257.* (**b**) The three-dimensional structure of *GmWD40-257*. The blue color represents protein regions without annotated domains. The green color indicates the FBOX domain. The purple color denotes the WD40 domain. (**c**) The schematic diagram of conserved domains for *Gmwd40-257.* (**d**) The three-dimensional structure of *Gmwd40-257*. The blue color represents protein regions without annotated domains. The green color indicates the FBOX domain.

To elucidate the function of *GmWD40*, we used a nonsense mutation (*Glyma.11g159230*, named *Gmwd40-257*), in which the 122nd amino acid tryptophan (W) was mutated to a premature termination codon, while the full-length of the wild-type protein consisted of 444 amino acids (Figure 1). The contents of protein, oil, fatty acids, soluble sugars, and tocopherol in the seeds were measured as follows:

The seed oil content was measured by the solvent extraction method [46]. In brief, put 250 mL of the flat-bottom flask and the thimble were placed in an oven at 105 ± 2 °C for 2 h, then transferred to a desiccator and cooled to constant weight. The flask was weighed, and the mass was recorded as m0. Then 3 g (±0.0001 g) of the ground soybean seed powder was accurately weighed into the thimble, and its mass was recorded as m1. Subsequently, the opening of the thimble was plugged with absorbent cotton. The thimble was placed into the flat bottom flask, and 150 mL petroleum ether was added. The mixture was then heated at a constant temperature of 70 °C to reflux. The process was continued for the duration of 12 h for each sample. At the end of each extraction process, the milled sample was removed from the thimble and the extraction process repeated for a different sample. After recovering the petroleum ether from the flat bottom flask, it was placed in a 105 °C oven to dry for 2 h. Then, it was put in a desiccator to cool down, and the flat bottom flask was weighed m2. The seed oil content was calculated by the formula: (m2 − m0)/m1 × 100%.

Seed protein content was determined by the Kjeldahl method with minor modifications [47]. Briefly, 0.2 g (±0.0001 g) of ground seed powder was digested with 5 mL H_2_SO_4_ and 4 g composite catalyst (CuSO_4_ and K_2_SO_4_) at 250 °C in a sterilizer for 30 min. After H_2_SO_4_ decomposed and emitted a large amount of white smoke, the temperature increased to 400 °C for 3 h. A blank test was conducted under similar conditions. Nitrogen content was determined using a FOSS Kjeltec 8400 instrument (Hillerød, Denmark).

Seed soluble sugar content was measured by the anthrone method [48]. Briefly, a 0.20% anthrone reagent (0.20 g anthrone dissolved in 100 mL H_2_SO_4_) and a 100 μg/mL glucose standard solution were prepared. The glucose standard solution (0.0, 0.2, 0.4, 0.6, 0.8, and 1.0 mL) was pipetted into separate test tubes, and distilled water was added to a final volume of 1.0 mL in each tube. Then, 4.0 mL of anthrone reagent was added to each tube and mixed rapidly. The tubes were heated in a boiling water bath for 10 min and rapidly cooled to room temperature. The absorbance of each solution was measured at 630 nm, and a standard curve was constructed. Grinded soybean powder (0.2 g ± 0.0001 g) was weighed into a tube, mixed with 5 mL of 80% ethanol, and incubated in a water bath at 80 °C for 30 min. After centrifugation (4000 r/min for 10 min), the supernatant was collected, and the extraction was repeated twice. The combined supernatants were transferred to a glass test tube. Then, 1.0 mL of the sample extract was mixed with 4.0 mL of anthrone reagent, heated in a boiling water bath for color development, and cooled. The absorbance was measured at 630 nm, and the total sugar content was calculated from the standard curve.

Soybean seed fatty acid contents were measured by a gas chromatography system (Agilent 7890A) as in previous studies [49]. Briefly, 30 mg of soybean ground powder was weighed into a 2 mL tube, and 1 mL of n-hexane was added. Then the mixture was incubated in a 60 °C water bath for 20 min. After incubation, 800 μL of sodium methoxide solution (1 mol/L) was added, and the mixture was vortexed for 10 min, followed by standing at 4 °C for 30 min. Subsequently, 600 μL of the supernatant was filtered through a 0.22 μm membrane and transferred to a 2 mL autosampler vial for instrumental analysis. The fatty acid content was determined using a gas chromatograph (Agilent Technologies, Palo Alto, CA, USA). The separation was performed on an Agilent DB-FastFAME column. The inlet temperature was set at 250 °C, and a split ratio of 50:1 was used. Helium was applied as the carrier gas under constant pressure mode at 14 psi. The injection volume was 1 μL. Oven temperature was set at 50 °C for 0.5 min, then gradually increased at the rate of 25 °C/min to 194 °C and held for 1 min, then increased at 5 °C/min to 245 °C and held for 3 min. The detector temperature was kept at 280 °C. The detector gas flows were adjusted to 40 mL/min for hydrogen, 400 mL/min for air, and 25 mL/min for the makeup gas.

Seed tocopherol content was measured by High-Performance Liquid Chromatography (HPLC) [49]. Briefly, 0.2 g of seed powder was weighed into a test tube. Subsequently, 0.05 g of ascorbic acid and 4 mL of 80% ethanol solution were added. The mixture was then subjected to ultrasonic extraction in a low-temperature water bath for 30 min, followed by the addition of 8 mL of n-hexane solution. The mixture was sonicated again in a low-temperature water bath for 30 min, centrifuged, and 2 mL of the supernatant was taken and dried under nitrogen. The residue was reconstituted with 0.5 mL of methanol and filtered through a 0.22 μm organic phase membrane. Tocopherols were resolved on a Poroshell EC-C18 column (4.6 × 100 mm, 2.7 μm), and the column temperature was maintained at 35 °C during the analytical process. The mobile phase comprised 80% acetonitrile and 20% water containing 20 mmol/L KH_2_PO_4_ (pH = 3.5) with a flow rate of 1.0 mL/min and a detection wavelength of 294 nm. Three duplicate measurements were performed for each sample. Standard curves were generated for each tocopherol (50, 100, 200, 250, 500, and 1000 ng/mL) using the same analytical method. The tocopherol standards were purchased from Shanghai Yuanye Bio-Technology Co., Ltd. (Shanghai, China), with a purity of ≥98%. Soybean seed tocopherol content was calculated based on standard curves.

### 2.9. Prediction of Interaction Between GmWD40-257 and Transcription Factor

The protein sequences of GmMYB311 and GmSL20 were obtained from Phytozome (https://phytozome-next.jgi.doe.gov/, accessed on 12 January 2026). AlphaFold 3 was subsequently applied to predict the protein-DNA complex. Then, the “.cif” file was imported into the PyMOL 3.0.3 software for hydrogen bond identification, RMSD quantification, structural visualization, and export of a PDB file for the protein monomers and multimeric complex [50,51].

### 2.10. Statistical Analysis

The statistical significance of the data was evaluated using a two-tailed Student’s *t*-test in EXCEL software. The plotting was implemented using GraphPad Prism version 9.0.

## 3. Results

### 3.1. Identification of GmWD40 Gene Family in Soybean

A genome-wide identification of *WD40* gene family members in soybean was performed using the latest reference genome (Wm82.a6.v1), resulting in the identification of 458 genes. Physicochemical analysis detected substantial variation among GmWD40 proteins. The lengths of GmWD40 proteins ranged from 106 to 3256 amino acids, with relative molecular weights ranging from 11,530.95 to 365,324.53 Da. Moreover, the GmWD40 proteins exhibited a wide isoelectric point (pI) distribution, spanning from 4.08 to 9.78, with 328 proteins demonstrating a pI below 7, suggesting that most GmWD40 proteins were acidic. Furthermore, among 458 GmWD40 proteins, 445 exhibited a negative hydropathy index, and only 13 showed a positive index, indicating that the majority of GmWD40 proteins were hydrophilic. The instability index of GmWD40 family members ranges from 20.41 to 70.76, with 180 members below 40 and the other 278 members at or above 40. In addition, the prediction of subcellular localization indicated that GmWD40 proteins were mainly localized in the nucleus, chloroplasts, and cytoplasm (Table S1).

### 3.2. Chromosome Distribution of GmWD40 in Soybean

Analysis of the chromosome distribution for the *GmWD40* gene family revealed that the 458 genes were distributed across the 20 chromosomes, with chromosome 10 containing the most genes (34), followed by chromosomes 8 and 13 (33 each), while chromosome 16 has the fewest (12). Additionally, the *GmWD40* genes exhibit clear clustering, with the majority located at the chromosome ends (Figure S1).

### 3.3. Analysis of Cis-Acting Elements for GmWD40

To investigate the potential regulatory mechanisms of *GmWD40*, the present study performed a cis-acting element analysis on promoter sequences (upstream 2000 bp). The results showed that the promoter regions of *GmWD40* contained a large number of regulatory elements (Figure S2). A total of 19 types of cis-acting elements with important functions were identified, including those involved in cell cycle regulation, auxin response, abscisic acid response, salicylic acid response, and abiotic stress response (Figure S2). These elements participate in multiple biological processes, including soybean growth and development regulation, hormone signaling, and biotic and abiotic stress responses. Most importantly, numerous cis-regulatory elements associated with soybean seed quality traits were identified, including ACCAAA, AAGTCA and CTCAAA. Therefore, we hypothesize that WD40 family genes may participate in the regulation of soybean quality-related traits. These findings suggest that *GmWD40* family members are co-regulated by multiple hormone signaling pathways, including GA, ABA, and salicylic acid, and that they play key roles in soybean seed development, plant growth and development, as well as biotic and abiotic stresses.

### 3.4. Phylogenetic Tree and Synteny Analysis

**Figure 2:**
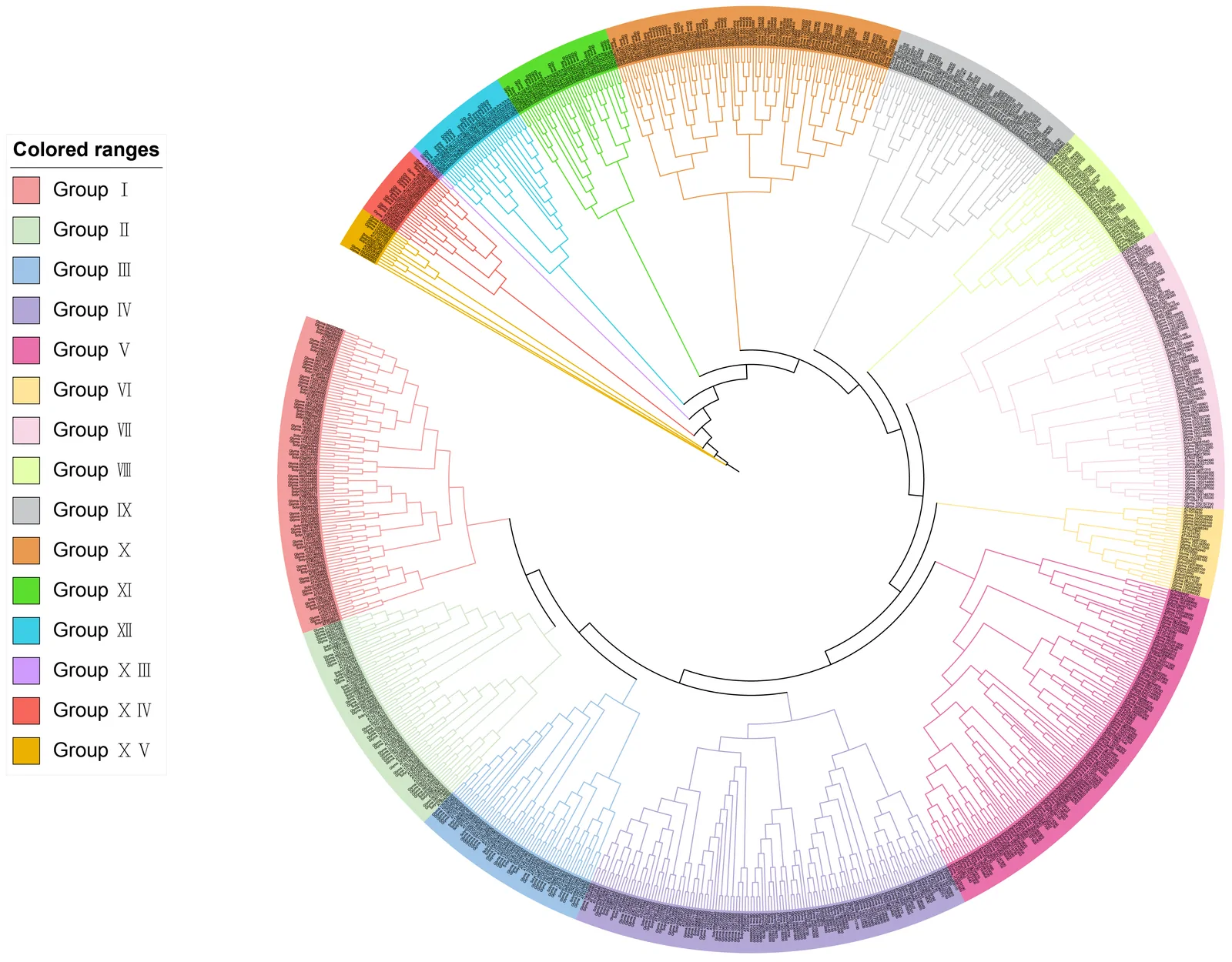
The phylogenetic tree and classification of GmWD40 in soybean. The different colors represent different groups.

To clarify the evolutionary relationships among GmWD40, tomato WD40 proteins and *A. thaliana* WD40 proteins, the present study constructed a phylogenetic tree using 458 soybean WD40 protein sequences, 207 tomato WD40 protein sequences and 230 *A. thaliana* protein sequences (Figure 2, Table S2). The results showed that these 895 proteins could be divided into 15 groups (Group I to Group XV), among which Group IV and V contained the largest number of WD40 (124), and Group XIII contained the fewest (2). Further analysis indicated that GmWD40s were mainly clustered within Groups I (10.48%), IV (13.97%), V (12.23%), VII (11.14%) and X (10.92%). The *A. thaliana* WD40s were also clustered within Groups I (10.87%), IV (13.91%), V (13.48%), VII (10.00%) and X (10.00%). While the tomato WD40s were also clustered within Groups I (9.66%), IV (14.49%), V (17.87%) and X (13.04%).

**Figure 3:**
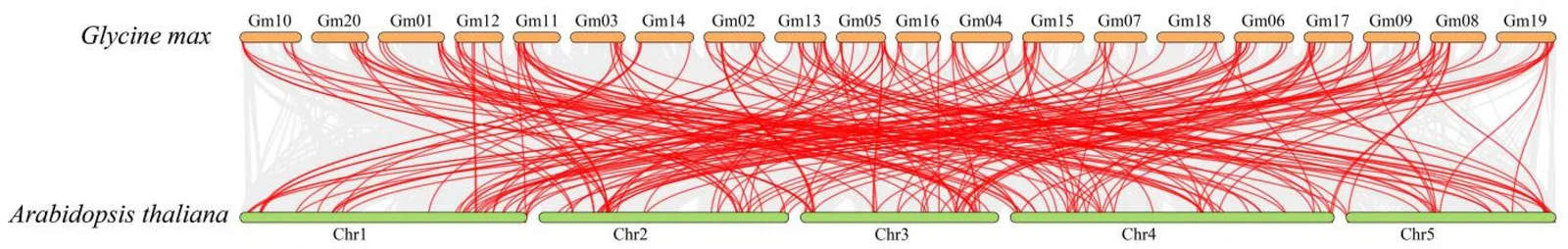
Synteny analysis of WD40 family genes in soybean and *A. thaliana*. The orange strips indicate soybean chromosomes, and the green strips represent chromosomes of *A. thaliana*. Gm01 to Gm20 correspond to the 20 soybean chromosomes, while Chr1 to Chr5 stand for the five chromosomes of *A. thaliana*. Red and gray lines both stand for homologous gene pairs between soybean and *A. thaliana*. Red lines correspond to WD40 homologous genes, while gray lines represent other homologous gene pairs.

To elucidate the homologous relationships between *GmWD40* and *A. thaliana WD40* genes, a synteny analysis was performed. The results revealed that 160 synteny genes were identified between soybean and *A. thaliana*, indicating that *WD40* genes were highly conserved between soybean and *A. thaliana* (Figure 3). Among these, *AtAGB1* (*AT4G34460*), involved in drought stress in *A. thaliana,* was homologous to *Glyma.11G118500* in soybean. Additionally, the *A. thaliana* anthocyanin biosynthesis-related gene *AT5G24520* was found to be homologous to *Glyma.06G136900* in soybean.

### 3.5. Analysis of Gene Structure and Conserved Motifs of GmWD40

Analysis of GmWD40 gene structures indicated considerable variation in gene length and intron number across different family members. For example, the longest gene, *Glyma.18G223300* (64,195 bp), contained 32 exons and 31 introns, indicating a relatively complex gene structure (Figure S3). Conserved motifs are the key structural elements underlying the core functions of a gene family. The conserved motifs of GmWD40 proteins were predicted via MEME (https://meme-suite.org/meme/tools/meme). A total of 10 conserved motifs (Motif 1~Motif 10) were identified, and the numbers of genes containing conserved Motifs 1 to 10 were 444, 438, 365, 409, 389, 265, 5, 379, 4, and 6, respectively (Figure S4).

### 3.6. Analysis of Interaction Network for GmWD40

The protein–protein interaction network of GmWD40 comprised 428 nodes and 491 interactions. The results showed that most interactions in the network have higher confidence scores (>0.95), indicating that these interactions are reliable (Figure S5). Further analysis revealed that Glyma.19G186600 was the key protein with the highest degree of connectivity in the network, followed by Glyma.01G001600, which interacted with multiple family members such as Glyma.20G190200, Glyma.17G233900, and Glyma.16G003700. This finding suggested that Glyma.19G186600 and Glyma.01G001600 might play a central role in the assembly of protein complexes or in signal transduction, integrating regulatory signals through interactions with multiple family members.

### 3.7. Expression Analysis of GmWD40 During Soybean Seed Development

**Figure 4:**
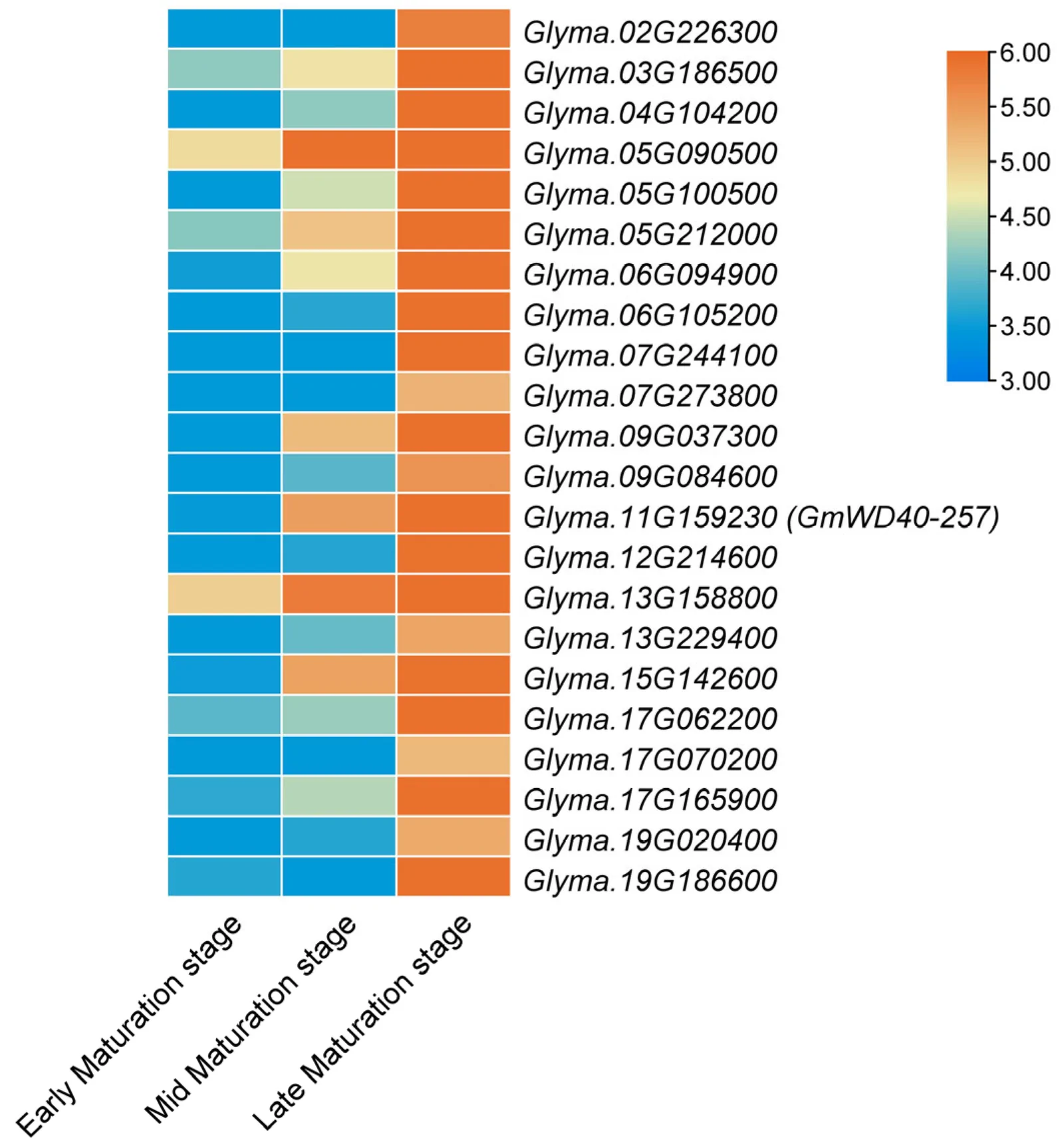
The differentially expressed genes of *GmWD* during seed development. The early maturation stage represents embryos isolated from seeds 6~7 mm in length; the mid maturation stage denotes embryos harvested from seeds weighing 200~250 mg, and the late maturation stage indicates embryos dissected from seeds weighing 230~500 mg. These data were derived from the NCBI GEO dataset GSE99571 (https://www.ncbi.nlm.nih.gov/geo/query/acc.cgi?acc=GSE99571, accessed on 12 January 2026).

Analysis of the promoter region revealed numerous cis-regulatory elements linked to soybean seed quality traits, such as ACCAAA, AAGTCA, and CTCAAA. Therefore, we profiled the expression of *GmWD40* across soybean seed development stages with published transcriptome data (GSE99571). The result showed that 22 differentially expressed genes were screened out. Among these, expression levels of sixteen genes, such as *Glyma.03G186500*, *Glyma.04G104200*, *Glyma.05G100500*, *Glyma.11G159230*, *Glyma.17G062200* and *Glyma.19G186600*, steadily increased throughout soybean seed development. Moreover, five candidate genes (*Glyma.02G226300*, *Glyma.07G244100*, *Glyma.07G273800*, *Glyma.17G070200*) were upregulated at the late developmental stage of soybean seed. In contrast, two genes (*Glyma.05G090500* and *Glyma.13G158800*) exhibited increased expression during the middle developmental stage (Figure 4).

### 3.8. GmWD40-257 Regulated Soybean Seed Quality

**Figure 5:**
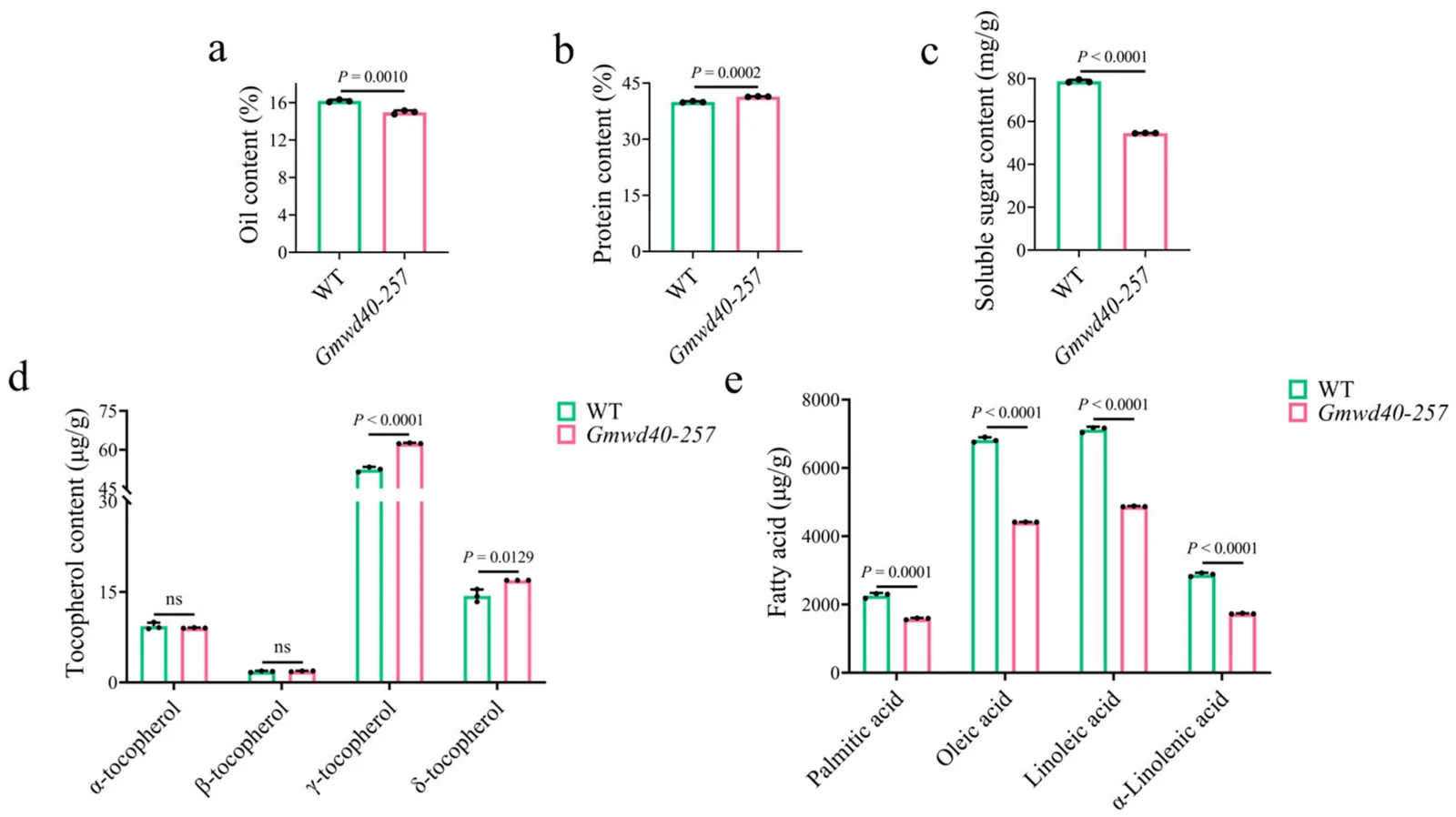
*GmWD40-257* regulated soybean seed quality. (**a**) Significant reduction in seed oil content for *Gmwd40-257*. (**b**) Significant increase in seed protein content for *Gmwd40-257.* (**c**) Significant decrease in seed soluble sugar content for *Gmwd40-257*. (**d**) Significant enhancement in seed γ and *δ* tocopherol content for *Gmwd40-257*. ns stands for no significance. (**e**) Significant decrease in fatty acid content for *Gmwd40-257*.

To elucidate the function of *GmWD40*, the nonsense mutation of *GmWD40-257* was employed to evaluate seed quality-related traits. The results showed that the seed protein content of *Gmwd40-257* was significantly increased by 3.56%, the γ-tocopherol content significantly increased by 19.26%, and the δ-tocopherol content significantly increased by 17.82%, while the α- and β-tocopherol contents showed no significant differences compared with WT (Figure 5 and Figure S6, Table S3). These findings indicated that *GmWD40-257* negatively regulated soybean protein, γ-tocopherol, and δ-tocopherol contents. Furthermore, the seed oil content of *Gmwd40-257* was significantly reduced by 7.50%, the palmitic acid decreased by 30.14%, oleic acid decreased by 35.27%, linoleic acid decreased by 31.64%, α-linolenic acid decreased by 39.71%, and soluble sugar content decreased by 30.76% (Figure 5, Table S3). These results suggested that loss of function of *GmWD40-257* affected the synthesis of seed oil, fatty acids, and soluble sugars in soybean.

### 3.9. Predict the Regulation of GmWD40-257 via AI

**Figure 6:**
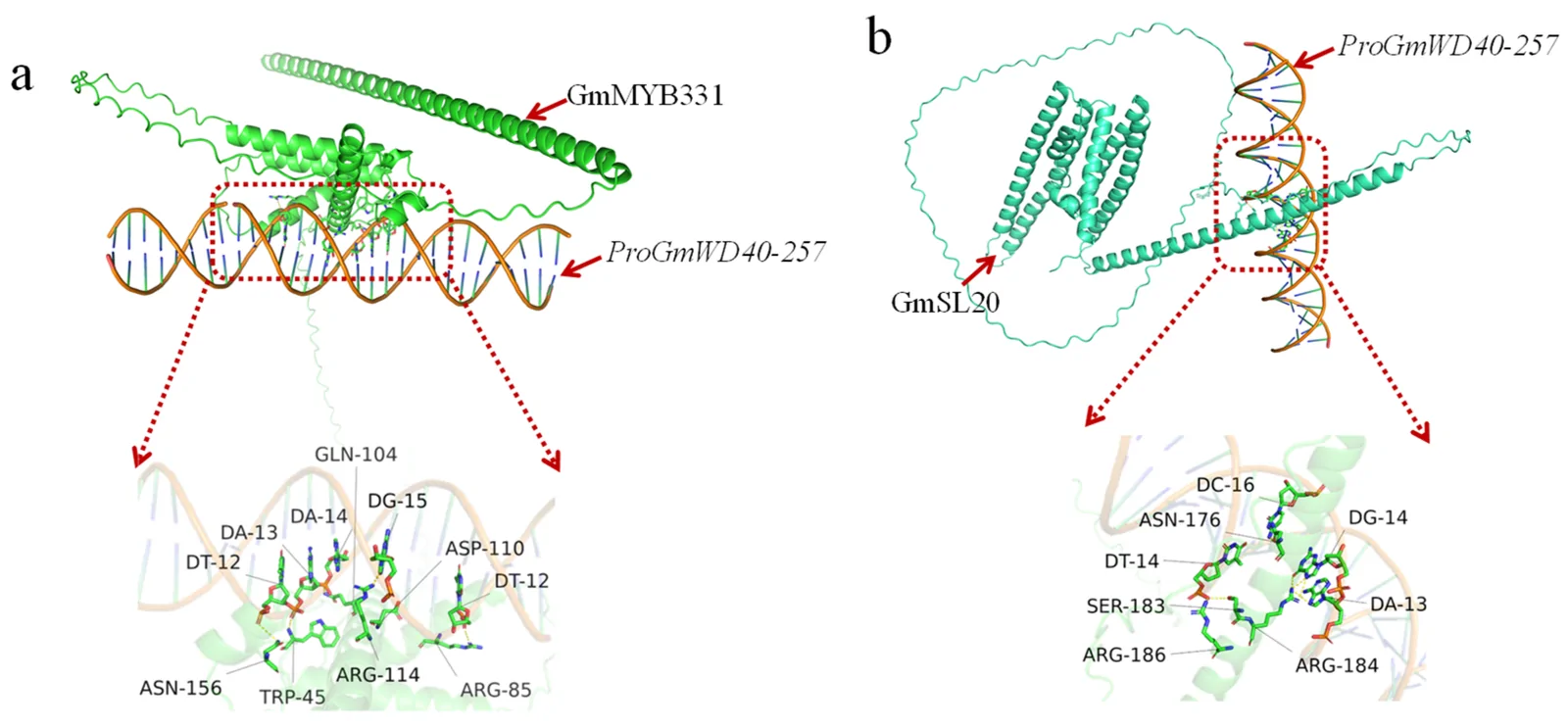
Prediction of the regulation of *GmWD40-257* by GmMYB331 and GmSL20. (**a**) The interaction between *GmWD40-257* and GmMYB331 predicted by the AlphaFold 3 method. Amino acid residues are designated by three-letter abbreviations, and the numeral following each amino acid denotes its position within the protein sequence. DNA bases are denoted by two-letter codes, where the second letter specifies the DNA base. (**b**) The interaction between *GmWD40-257* and GmSL20 via the AlphaFold 3 method. Amino acid residues are designated by three-letter abbreviations, and the numeral following each amino acid denotes its position within the protein sequence. DNA bases are denoted by two-letter codes, where the second letter specifies the DNA base.

Based on the results of cis-acting element analysis and functional validation, we hypothesized that *GmWD40-257* was regulated by multiple transcription factors associated with soybean seed quality traits. Previous studies have identified that GmMYB331 and GmSL20 (Seed Length 20) were important regulators for oil or fatty acid content and seed size in soybean. Therefore, we speculated that these two factors may interact with the promoter of *GmWD40-257*. To further explore the regulatory mechanism of *GmWD40-257* in modulating soybean seed quality, AlphaFold 3 was used to predict its protein-DNA interactions. The results revealed that six amino acid residues of GmMYB331 and five DNA bases of the *GmWD40-257* promoter were involved at the binding interface, where five hydrogen bonds were formed (Figure 6). Further analysis indicated that among six amino acid residues, five amino acid residues (except asparagine) resided within the conserved domain of GmMYB331. Similarly, four amino acid residues of GmSL20 and four DNA bases of the *GmWD40-257* promoter mediated binding, with five hydrogen bonds generated in this interaction as well. Meanwhile, these four amino acid residues were mapped to the bzip domain of GmSL20 (Figure 6).

## 4. Discussion

WD40 proteins function as multifunctional molecular scaffolds to mediate protein–protein interactions and participate in diverse biological pathways [52,53,54,55,56]. The *WD40* gene family varies in member number across plant species. In this study, a total of 458 *WD40* genes were identified in soybean. By comparison, the gene copy number of the WD40 family is 207 in tomato [57], 743 in wheat [58], 200 in rice [59], 278 in carrot [60], 579 in cotton [61], and 230 in *A. thaliana* [9]. Furthermore, physicochemical property analysis uncovered variations across GmWD40 family members in protein length, molecular weight and isoelectric point. Such divergence implied that these proteins may execute diverse functions under different biological conditions. Moreover, among the 458 GmWD40 proteins, 278 were classified as unstable proteins, suggesting their potential involvement in the regulation of stress responses in soybean [62,63]. Chromosomal distribution analysis showed that GmWD40 proteins exhibit a certain clustering on soybean chromosomes, with most genes distributed at the ends of chromosomes, which is consistent with a previous report [58].

Synteny analysis identified 160 WD40 homologous genes between the soybean and *A. thaliana*. Among them, *AtAGB1* (*AT4G34460*) was homologous to *Glyma.11G118500* in soybean. In *A. thaliana*, *AtAGB1* was involved in drought stress signal response, and its loss-of-function mutant *agb1-2* showed reduced leaf water loss, lower stomatal length and density, and enhanced drought tolerance, implying that the soybean ortholog *Glyma.11G118500* may function analogously in drought stress response [64]. Additionally, *AT5G24520* exhibited high synteny with the soybean gene *Glyma.06G136900*. In *A. thaliana*, *AT5G24520* is known to regulate anthocyanin accumulation, and its mutants lacked purple anthocyanin pigments, resulting in a transparent seed coat through which the yellow cotyledons are visible. This functional characterization suggested that *Glyma.06G136900* might similarly play a positive role in anthocyanin biosynthesis in soybean [65]. Cis-acting elements play important roles in the regulation of gene transcription. Promoter analysis of *GmWD40* detected various cis-acting elements, including GA-responsive elements, ABA-responsive elements, and defense/stress-related elements, suggesting that *GmWD40* may achieve functional regulation by participation in different signaling pathways. The endogenous plant hormone GA plays a key role in plant growth and development processes such as stem elongation, pollen maturation, and flowering. It also interacts with other hormones, including ABA, auxin (IAA), jasmonic acid (JA), ethylene (ET), and cytokinin (CTK). These hormonal interactions are closely associated with plant growth, development, and stress tolerance [66,67].

WD40 family genes were involved in seed development in many crops. Loss-of-function of the rice WD40 protein gene *DRW1* leads to abnormally loose starch granules and premature cellularization in grains, resulting in shriveled rice kernels and severe yield loss. Conversely, overexpression of the full-length coding sequence of *DRW1* rescued the grain-filling phenotype of *drw1* plants. Mechanistic analysis revealed that *DRW1* regulated downstream gene expression by controlling the dynamic distribution of three epigenetic modifications—DNA 6mA, RNA m5C, and H3K27me3—thereby influencing rice yield and salt tolerance [68]. Mutation of the rice WD40 domain protein gene *GORI* results in reduced pollen germination rate and arrested pollen tube growth. Further mechanistic study showed that GORI (Germinating Modulator of Rice Pollen) functions as a scaffold protein in clathrin-mediated endocytosis (CME) [69]. During soybean seed development, proteins, oil, and other substances were synthesized and stored. When the seed germinated, these reserves were mobilized to provide energy for germination and seedling establishment. In addition to regulating seed development, the present study identified that *WD40* regulated seed quality. Loss function of *GmWD40-257* increased protein, γ-tocopherol, and δ-tocopherol content, while decreasing oil, palmitic acid, oleic acid, linoleic acid, α-linolenic acid, and soluble sugar contents in soybean seed. These results indicate that *GmWD40-257* positively regulated the content of oil, palmitic acid, oleic acid, linoleic acid, α-linolenic acid, and soluble sugar, and negatively regulated the contents of protein, γ-tocopherol, and δ-tocopherol. *GmWD40-257* might influence the synthesis of protein, oil and tocopherol by modulating carbon-nitrogen partitioning, expression of key enzymes, and transcription factor networks in soybean. Moreover, it has been reported that γ- and δ-tocopherols content in soybean seed was significantly positively correlated with oil content and significantly negatively correlated with protein content, whereas α-tocopherol showed a weak correlation with protein and fat contents [70]. The present study also found that the nonsense mutant *Gmwd40-257* exhibited significantly increased protein, γ-tocopherol, and δ-tocopherol contents, while significantly reduced oil content, and no significant change in α-tocopherol. Furthermore, previous studies have documented that GmMYB311 specifically binds to the ACCAAA motif, whereas GmSL20 targets the AAGTCA motif [71,72]. The promoter sequence of *GmWD40-257* harbors both ACCAAA and AAGTCA binding motifs. With AlphaFold3, we performed predictions of physical interactions between the *GmWD40-257* promoter and transcription factors GmMYB311 and GmSL20. These findings indicated that GmMYB311 and GmSL20 were likely functional regulators of *GmWD40-257*. In summary, *GmWD40-257* played a key regulatory role in the accumulation of protein, oil, tocopherol, and other quality traits in soybean seed, and it may be modulated by two transcription factors, GmMYB311 and GmSL20.

## 5. Conclusions

A total of 458 GmWD40 family genes were identified, which were distributed across 20 chromosomes. Phylogenetic analysis classified GmWD40 into eight groups. Synteny analysis identified 160 syntenic genes between soybean and *A. thaliana*. Conserved motif analysis detected ten core conserved motifs, among which Motif 1 and Motif 2 were the most widely observed. Cis-acting element analysis identified 19 types of cis-acting elements involved in growth and development, hormone responses, and stress responses. The protein–protein interaction network comprised 428 nodes and 491 high-confidence interactions. Functional analysis showed that the WD40 family member *GmWD40-257* positively regulated the content of oil, palmitic acid, oleic acid, linoleic acid, α-linolenic acid, and soluble sugars in soybean, while negatively regulating the contents of protein, γ-tocopherol, and δ-tocopherol.

## Data Availability

All data generated or analyzed during this study are included in this article and its Supplementary Information Files.

## Supplementary Materials

The following supporting information can be downloaded at: https://www.mdpi.com/article/10.3390/genes17080904/s1, Table S1: Information of WD40 family genes in soybean; Table S2: Classification and grouping of WD40 proteins from soybean, tomato and *A. thaliana* based on phylogenetic tree; Table S3: Profiles of soybean seed quality in WT and mutants; Figure S1: Chromosome distribution of *GmWD40* in soybean; Figure S2: Cis-acting elements analysis of *GmWD40* in soybean; Figure S3: Gene structure analysis of *GmWD40* in soybean; Figure S4: Motif structure analysis of GmWD40 in soybean; Figure S5: Protein interaction network of GmWD40 in soybean; Figure S6: Chromatograms of tocopherol standard and tocopherol extracted from soybean seeds.

## Abbreviations

The following abbreviations are used in this manuscript:

ABA	Abscisic acid
CME	Clathrin-mediated endocytosis
CTK	Cytokinin
ET	Ethylene
GA	Gibberellin
GH	Glycine-histidine
HPLC	High-performance liquid chromatography
IAA	Indole-3-acetic acid
JA	Jasmonic acid
ML	Maximum likelihood
pI	Isoelectric point
RUP	Repressor of UV-B photomorphogenesis
WD	Tryptophan-aspartate
WT	Wild type
