# Supplementary material for: Genome-Wide Identification of the WD40 Gene Family and Functional Analysis of a Candidate Gene Regulating Seed Quality in Soybean
Source: Genes (Basel). 2026 Jul 30;17(8):904. doi: 10.3390/genes17080904 (PMC13512697; doi:10.3390/genes17080904)
Supplement: Supplementary file 1 [file genes-17-00904-s001.zip › Supplement figure.pdf]

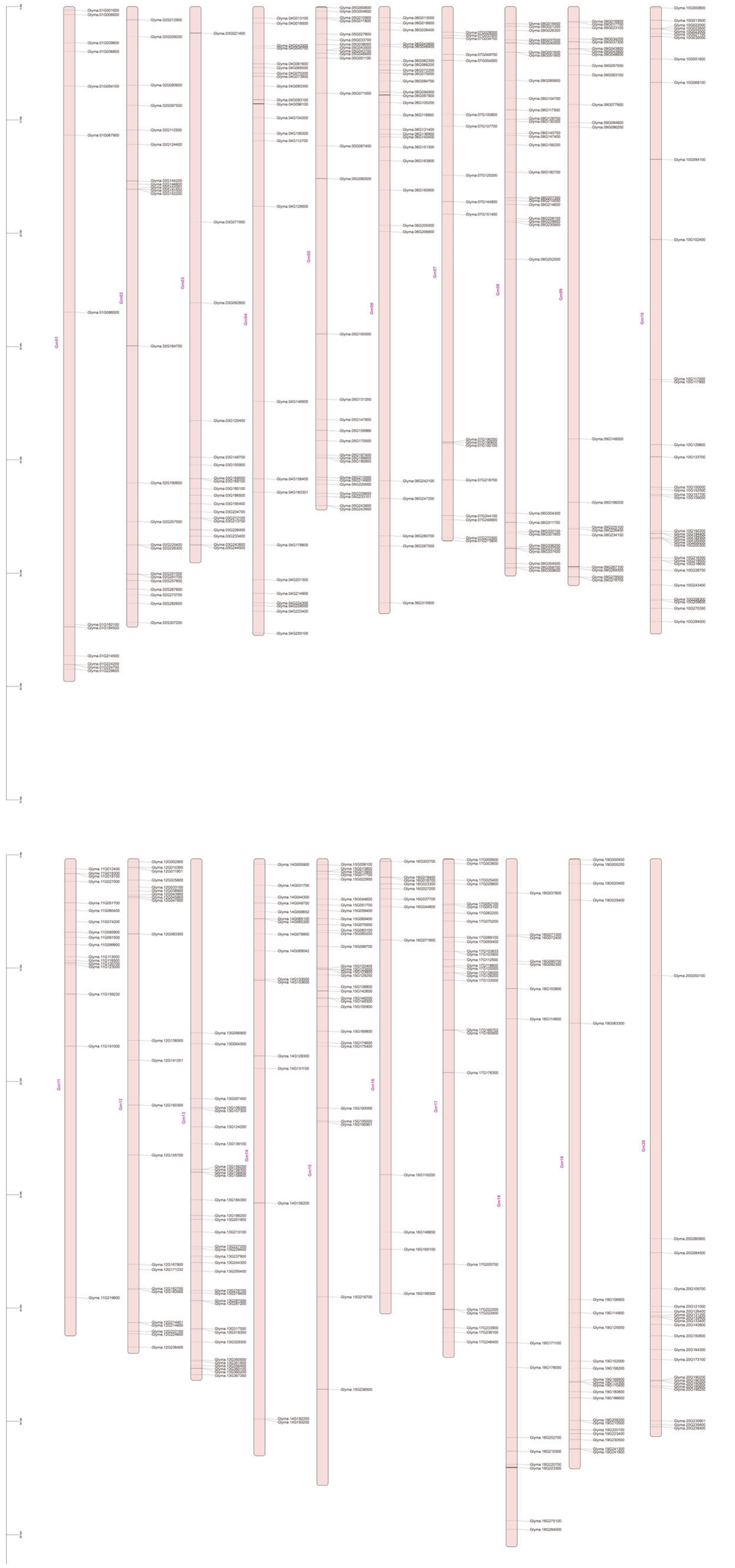

Figure S1. Chromosome distribution of *GmWD40* in soybean.

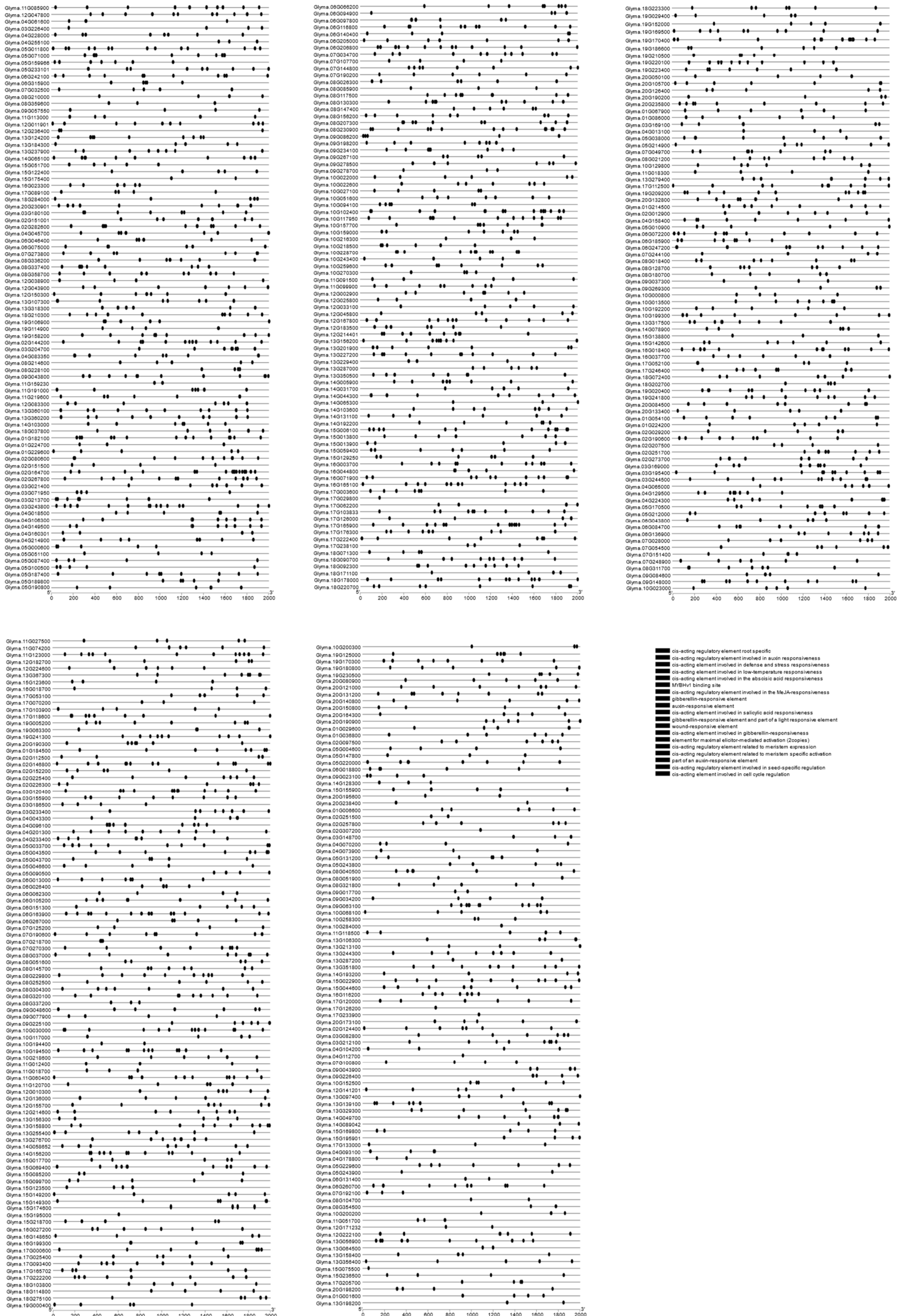

Figure S2. Cis-acting elements analysis of *GmWD40* in soybean.

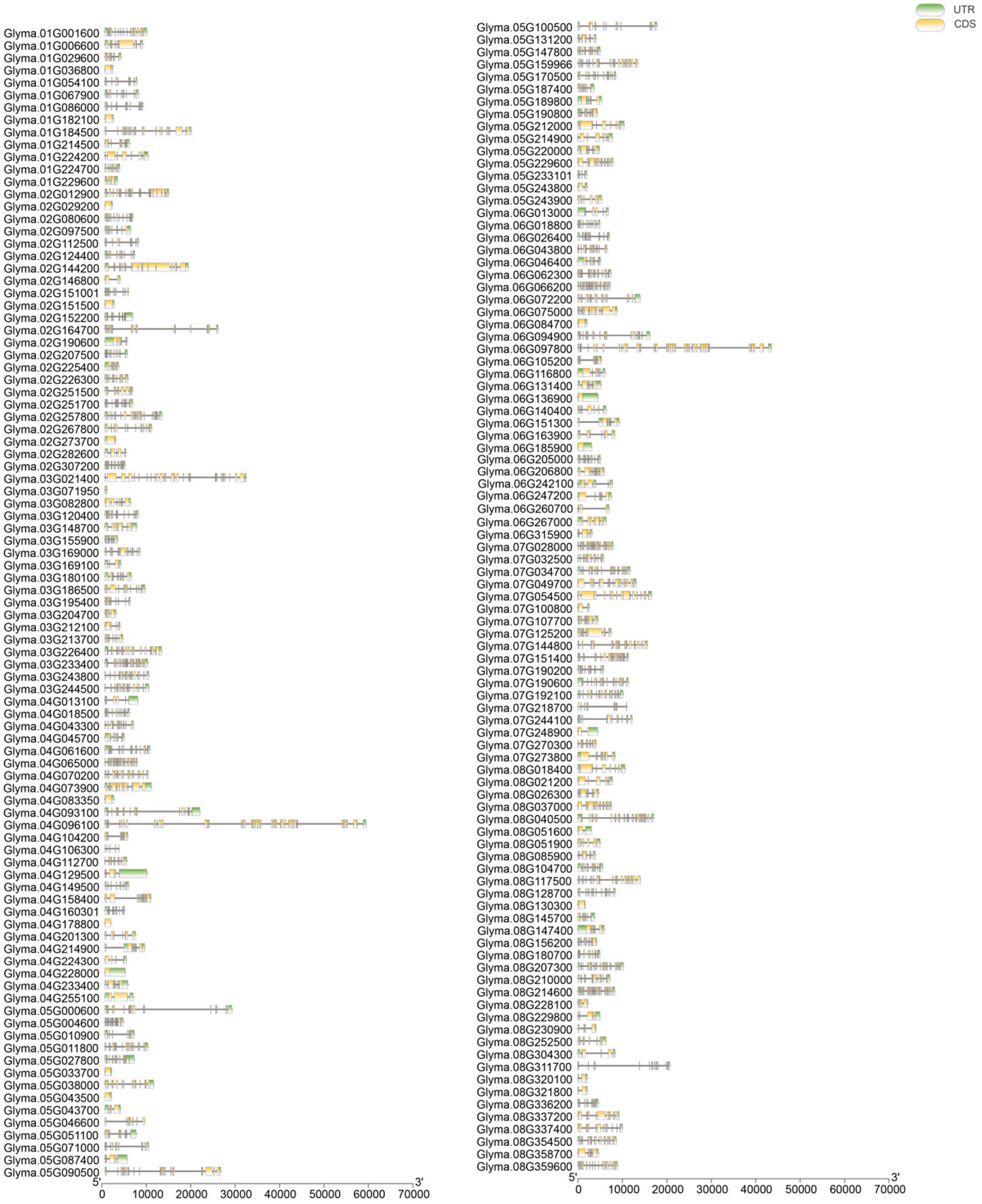

Figure S3. Gene structure analysis of *GmWD40* in soybean.

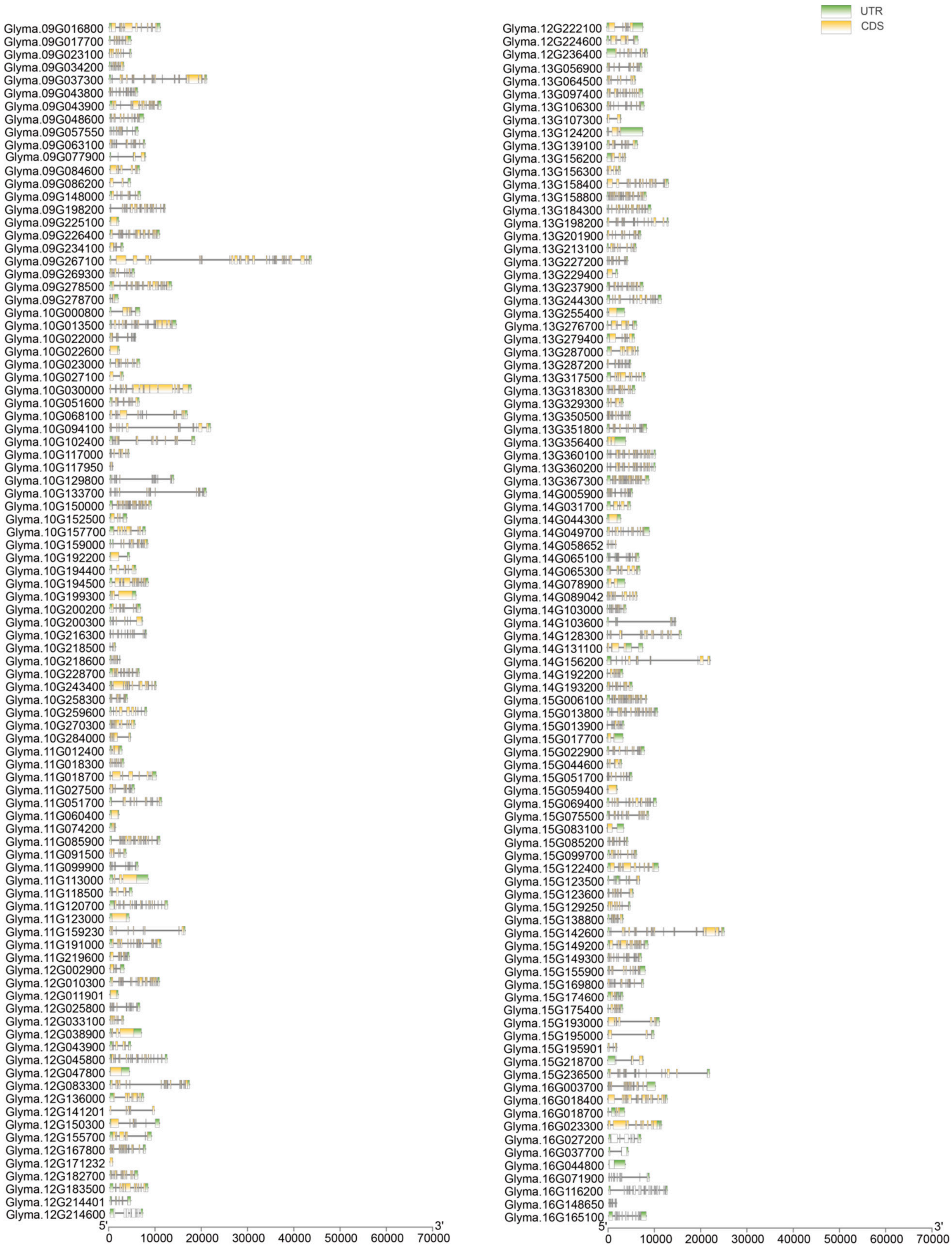

Figure S3. Gene structure analysis of *GmWD40* in soybean (continued).

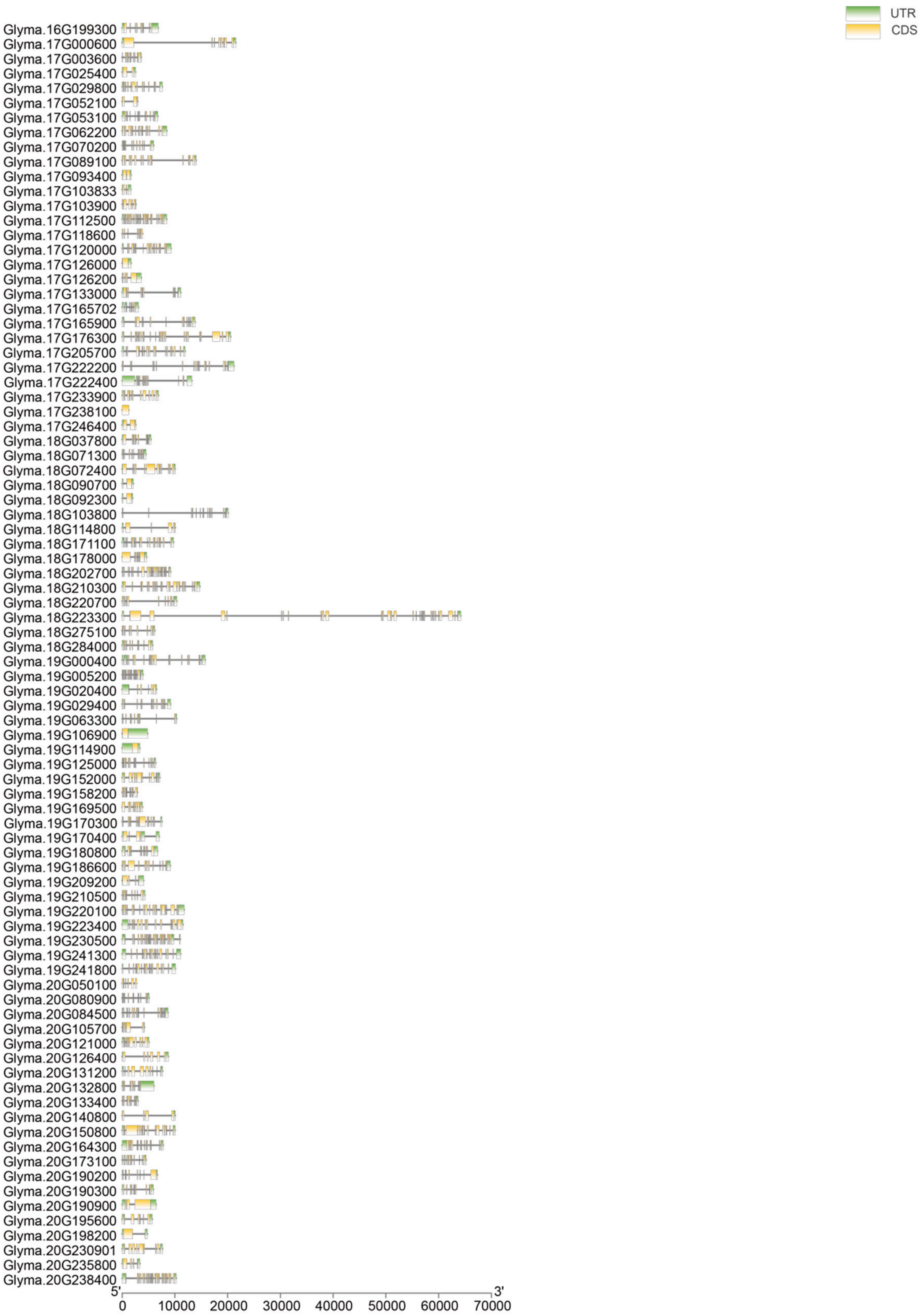

Figure S3. Gene structure analysis of *GmWD40* in soybean (continued).

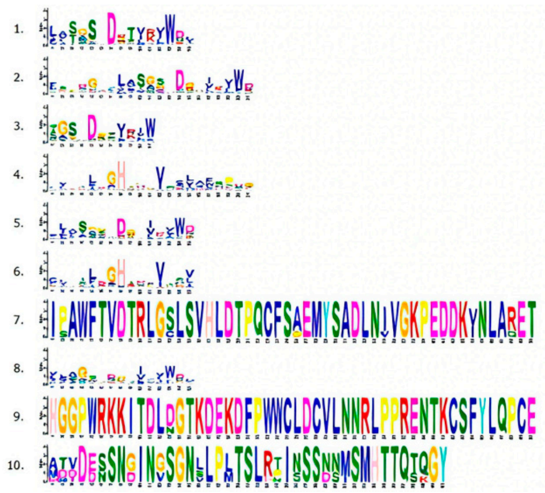

Figure S4. Motif structure analysis of GmWD40 in soybean.

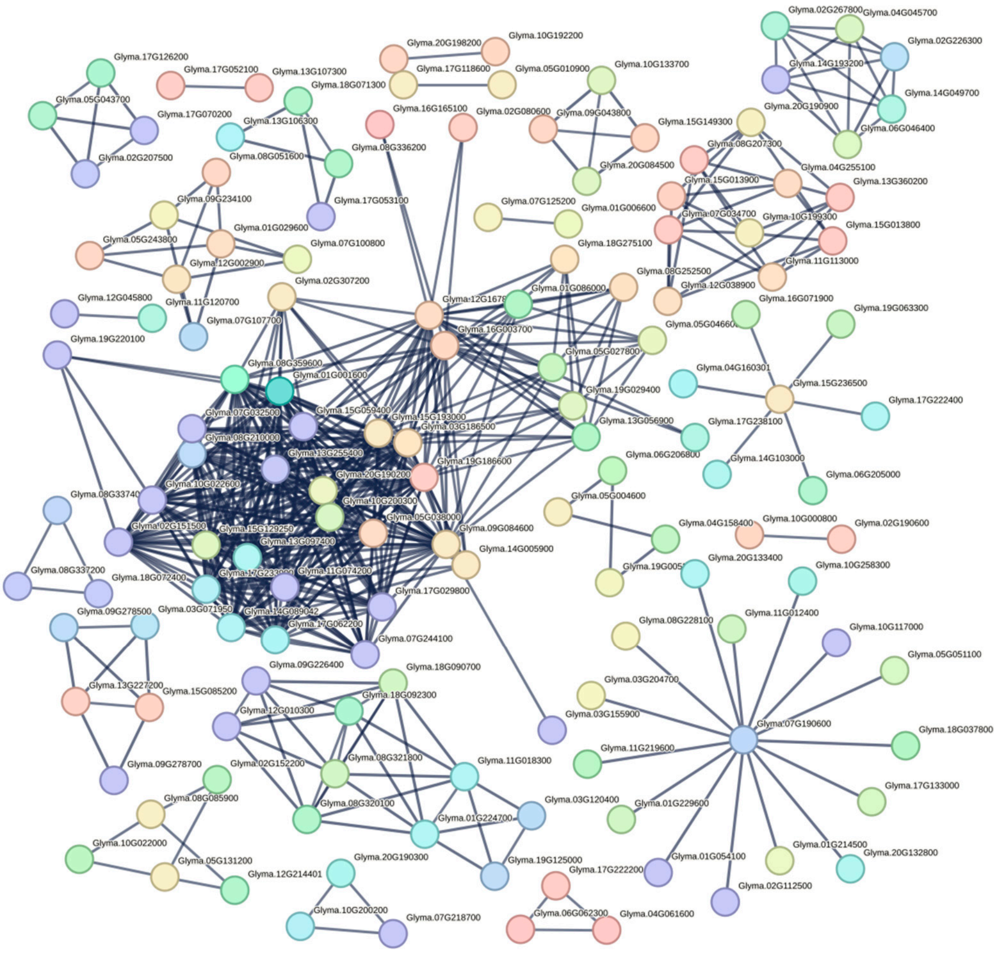

Figure S5. Protein interaction network of GmWD40 in soybean.

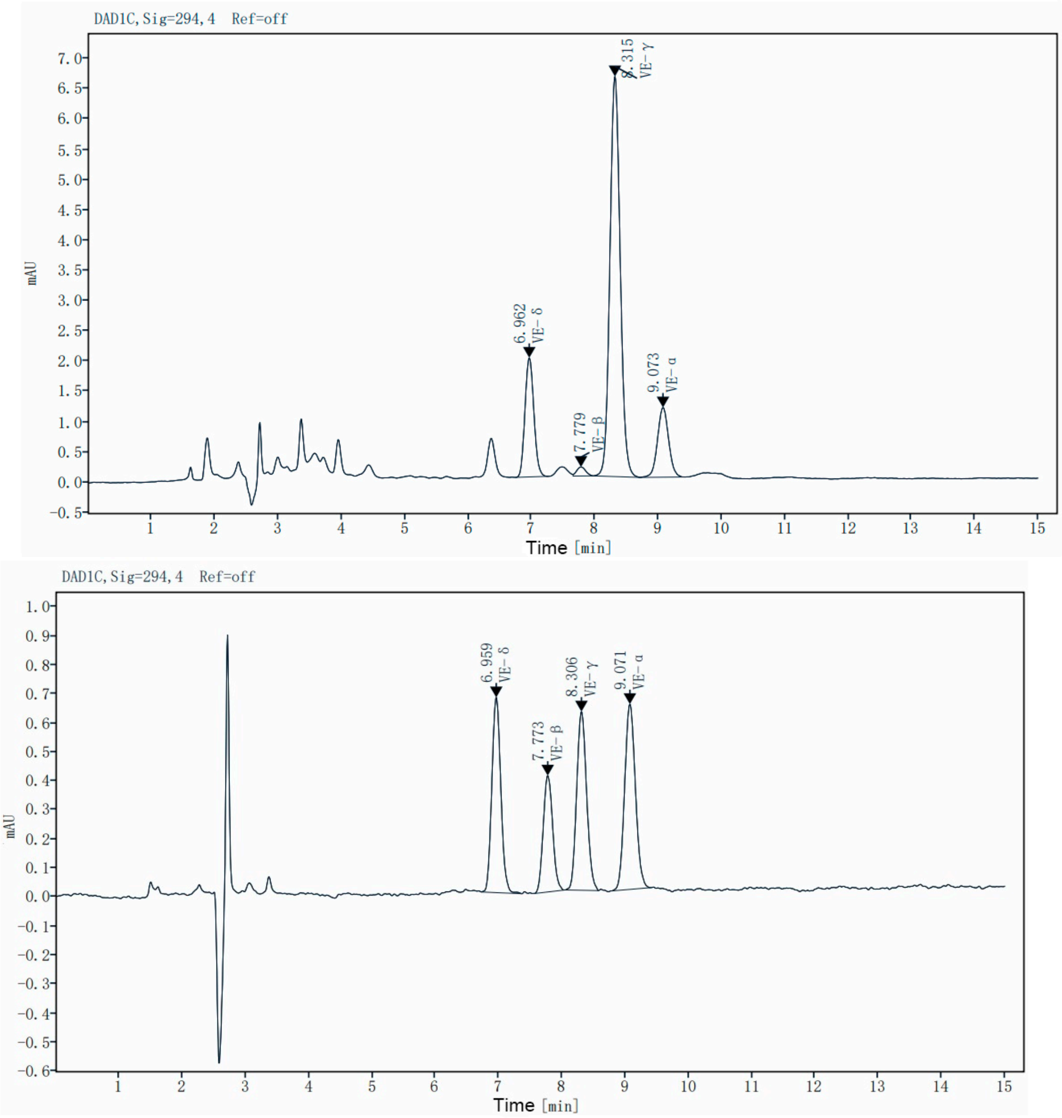

Figure S6. Chromatograms of tocopherol standard and tocopherols extracted from soybean seeds.
